# Initial-Care Medical and Prescription Costs for Incident Metastatic versus Nonmetastatic Colorectal Cancer

**DOI:** 10.1158/2767-9764.CRC-25-0367

**Published:** 2025-10-20

**Authors:** Chi M. Nguyen, Paul G. Yeh, Mai P. Nguyen, Travis S. Johnson, Hyunwoo Koo, Victoria L. Champion, Todd C. Skaar, David R. Lairson, Patrick O. Monahan, Thomas F. Imperiale, Hongmei Nan

**Affiliations:** 1 Department of Biostatistics & Health Data Science, Indiana University School of Medicine, Indianapolis, Indiana.; 2 School of Engineering Medicine, Texas A&M Naresh K. Vashisht College of Medicine, Bryan, Texas.; 3 Department of Management, Policy, and Community Health, University of Texas Health Science Center at Houston, School of Public Health, Houston, Texas.; 4 Asia Pacific Research Center, Freeman Spogli Institute for International Studies, Stanford University, Stanford, California.; 5 Division of Clinical Pharmacology, Department of Medicine, Indiana University School of Medicine, Indianapolis, Indiana.; 6 Department of Pharmacy Practice, Purdue University College of Pharmacy, West Lafayette, Indiana.; 7 Indiana University Melvin and Bren Simon Comprehensive Cancer Center, Indianapolis, Indiana.; 8 Indiana University School of Nursing, Indianapolis, Indiana.; 9 Department of Management, Policy, and Community Health, Center for Health Services Research, University of Texas Health Science Center at Houston, School of Public Health, Houston, Texas.; 10 Division of Hematology/Oncology, Department of Medicine, Indiana University School of Medicine, Indianapolis, Indiana.; 11 Department of Epidemiology, Richard M. Fairbanks School of Public Health, Indiana University, Indianapolis, Indiana.

## Abstract

Colorectal cancer is a leading cause of cancer-related death and among the costliest cancers to treat in the United States. As advanced-stage diagnoses increase, the initial-care cost differences between metastatic (mCRC) and nonmetastatic colorectal cancer (non-mCRC) remain unclear, limiting insights into the financial impact of metastasis and late detection. Using Optum’s de-identified Clinformatics Data Mart, the largest US commercial claims database, we examined first-year costs of care for patients aged ≥45 years newly diagnosed with colorectal cancer between 2017 and 2023, with at least 1 year of medical records before and after diagnosis. Multivariate linear mixed-effects models estimated the impact of metastasis on medical and prescription charges and out-of-pocket expenses (2023 USD) in relation to treatment components, emergency department (ED) visits, hospitalizations, and hospice care. Among 25,169 patients, 32.8% had metastasis at diagnosis. Treatment and care utilization were consistently higher for patients with mCRC, with the greatest disparity in pharmacotherapy use (79.1% vs. 26.9%), followed by ED visits (65.4% vs. 48.1%), hospitalizations (91.1% vs. 84%), radiotherapy, and hospice care. Medical and prescription charges for patients with mCRC ($353,255 and $5,745) were nearly double than those for non-mCRC ($182,000 and $2,406), as were out-of-pocket expenses ($4,032 vs. $2,144 for medical; $319 vs. $188 for prescriptions). Pharmacotherapy was the primary cost driver in mCRC, followed by hospitalizations and ED visits. Initial-care medical and prescription costs were substantially higher for patients with mCRC. These findings underscore the economic burden of metastasis, highlighting potential benefits and cost savings of early detection and intervention strategies such as routine screening.

**Significance:**

This study highlights the substantial economic burden of mCRC, with medical and prescription costs nearly twice those of nonmetastatic cases. The findings underscore the financial and clinical benefits of early detection and timely intervention, reinforcing the importance of routine colorectal cancer screening to reduce late-stage disease incidence.

## Introduction

Colorectal cancer is the second leading cause of cancer-related deaths and the third most commonly diagnosed cancer among both men and women in the United States (1, 2). It also represents the second-highest cancer-related treatment costs, accounting for 11.6% of total national cancer healthcare spending (3). When colorectal cancer progresses to a distant stage, which is referred to as metastatic colorectal cancer (mCRC), without timely medical intervention, patients require more aggressive treatments, face increased risks of complications, and experience significantly shorter survival compared with those diagnosed at earlier stages (4–8). Consequently, the medical costs associated with mCRC are substantially higher than those for non-advanced colorectal cancer (9–12). Early detection and effective treatment during the initial-care phase, the first year following diagnosis, are therefore crucial for improving outcomes and reducing the financial burden on healthcare system and patients.

Although colorectal cancer incidence has declined since the mid-1990s because of widespread screening (13–15), recent trends indicate a concerning increase in advanced-stage diagnoses, particularly among individuals under 65 years of age (2, 16–20). This shift places increased strain on healthcare systems and escalates medical costs for both providers and patients. Notably, there are limited data comparing the financial burden of mCRC with nonmetastatic colorectal cancer (non-mCRC) during the initial phase of care. Although some studies have assessed overall mCRC costs (10, 21, 22), few have dissected differences in treatment components, care settings, and other cost drivers between mCRC and non-mCRC. Understanding these distinctions is crucial for informing healthcare policy, clinical strategies, and resource allocation. Therefore, this study aims to quantify the cost differences between mCRC and non-mCRC during the initial-care phase and to identify key cost drivers influencing these differences.

## Materials and Methods

### Data and study design

We conducted a retrospective cohort study using de-identified, Health Insurance Portability and Accountability Act–compliant data from Optum’s Clinformatics Data Mart (CDM), provided by Indiana University (IU) Data Resources. Optum CDM is derived from a database of administrative health claims for members of large commercial and Medicare Advantage (MA) health plans, covering more than 75 million individuals in total across 50 US states spanning from January 2007 to December 2023 at the time of this study. The dataset included individual demographics, household income, health insurance types, pharmacy prescriptions, medical diagnoses, procedures, and service and facility charges, as well as associated copayments, deductibles, and coinsurance throughout individuals’ enrollment period (23, 24).

Data from January 1, 2016, to its latest available date of December 14, 2023, along with diagnosis codes from the International Classification of Diseases, 10th Revision, Clinical Modification (ICD-10-CM), were used to identify colorectal cancer new cases diagnosed between January 1, 2017, and November 30, 2022, which allowed identification of patients’ preexisting comorbidity at least 1 year prior to the diagnosis, as well as capture of patients’ care costs in 1 year after the diagnosis. Following guidelines of the US Armed Forces Health Surveillance Division on colorectal cancer case definition and incidence rules, ICD-10-CM codes *C18.x*, *C19.x*, *C20*, and *C26.0* with treatment-encountering codes *Z51.0* and *Z51.1* were applied to identify new colorectal cancer cases, with the first colorectal cancer encounter date serving as the incident date (Supplementary Table S1; ref. 25).

**Figure 1.**
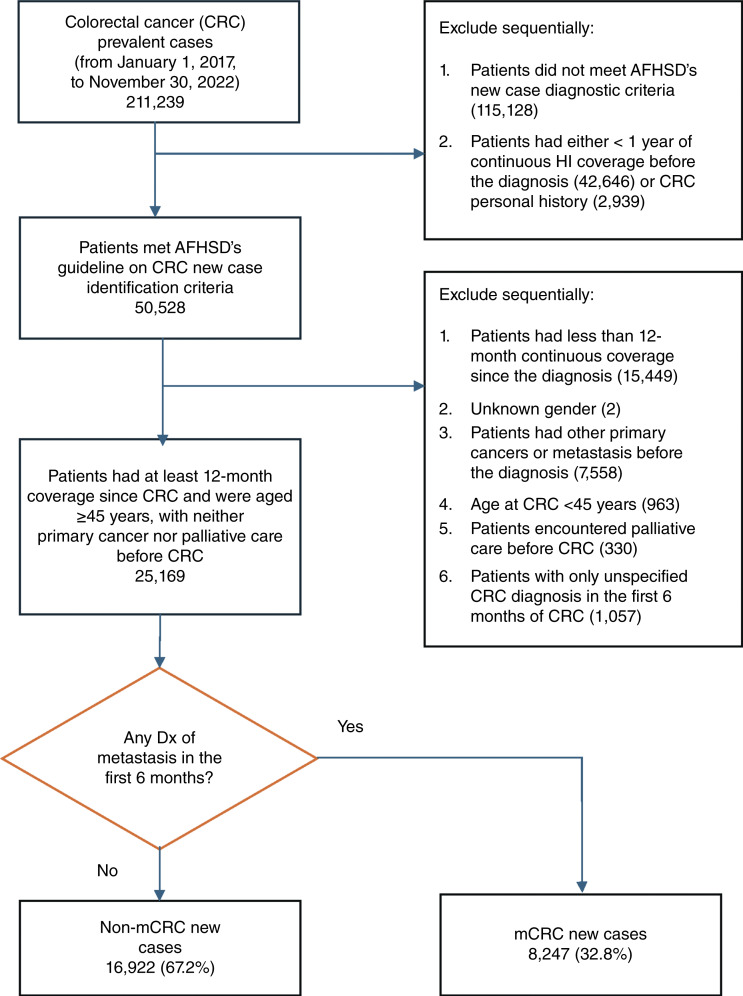
Derivation of new colorectal cancer (CRC) cases with and without metastasis. Flow diagram presenting steps to extract newly diagnosed colorectal cancer cases and to classify into nonmetastasis and metastasis incidences. AFHSD, The Armed Forces Health Surveillance Division (a partner with the Centers for Disease Control and Prevention on health surveillance and matters).

In accordance with the US Centers for Disease Control and Prevention’s guidelines recommending colorectal cancer screening beginning at the age of 45 years for average-risk individuals (effective in May 2021; ref. 15), our study included patients aged 45 years and older. Eligibility criteria required that patients have (i) at least 1 year of continuous insurance coverage before diagnosis, allowing for assessment of recent medical history; (ii) no prior diagnosis of other primary invasive cancers to ensure colorectal cancer was not secondary; (iii) a specified colorectal cancer diagnosis code within the first 6 months to identify newly diagnosed metastatic cases (26, 27); and (iv) continuous insurance coverage for 1 year after diagnosis, enabling calculation of complete initial-care costs. Patients were then grouped into those with metastasis (mCRC group) and those without (non-mCRC group). The cohort derivation is shown in Fig. 1.

### Outcome variables

The costs were divided into two categories: (i) medical service expenses, including healthcare professional services, facility use, laboratory and diagnostic testing, imaging, procedures, care visits, and cancer drug administration in which drug and delivery costs were billed together (Supplementary Table S2), and (ii) prescription expenses, covering pharmacy-dispensed outpatient medications. These costs, adjusted to 2023 USD using the US Consumer Price Index for Medical Care (https://fred.stlouisfed.org/series/CPIMEDSL), were analyzed from both the provider’s and patient’s perspectives. From the provider’s perspective, costs were measured by charges for medical services and medications, whereas from the patient’s perspective, costs were calculated by out-of-pocket (OOP) expenses comprising deductibles, copays, and coinsurance. Specifically, outcome variables included total medical charges, medical OOP expenses, prescription charges, and prescription OOP expenses during the initial-care phase, along with their relative changes compared with the year prior to the colorectal cancer incident.

### Cancer treatments, cost drivers, and other covariates

Cancer treatments were classified into four core components, with patients potentially receiving various combinations: laboratory and nonoperative diagnostic tests, ambulatory gastrointestinal (GI) surgery (excluding dental procedures), cancer pharmacotherapies (including chemotherapy, targeted therapy, immunotherapy, and their possible combinations), and therapeutic radiation. These components were identified using medical service types in insurance claims, along with Current Procedural Terminology and Healthcare Common Procedure Coding System codes (Supplementary Table S3).

Given the complexity of treating mCRC (5–7), complications may arise that require intensive or specialized care. For instance, major colorectal resections or aggressive treatments like metastasectomy for liver or lung metastases, specific to mCRC, typically require hospitalization and inpatient care. Accordingly, we captured initial-phase costs associated with emergency department (ED) visits, hospitalization, and hospice care. These care encounters were identified using service types in insurance claims (Supplementary Table S4). Additionally, common prescribed drugs for cancer, including antineoplastic agents, opioids, and antibiotics, were identified using the American Hospital Formulary Service Pharmacologic-Therapeutic Classification (Supplementary Table S5).

Other factors considered as cost drivers included patient demographics (e.g., gender, race/ethnicity, diagnosis age, and diagnosis year), household income, preexisting comorbidities, and insurance types. As comorbidities can significantly affect medical costs (28), we included all diagnosis codes prior to colorectal cancer diagnosis and utilized the Elixhauser Comorbidity Software refined for ICD-10-CM from the Healthcare Cost and Utilization Project (https://hcup-us.ahrq.gov/toolssoftware/comorbidityicd10/comorbidity_icd10.jsp#down) to calculate the Elixhauser Comorbidity Index (ECI), which was then categorized into four quartiles. Insurance types were classified as commercial health insurance, MA without Medicaid/Low-Income Subsidy (LIS), MA with Medicaid/LIS, and unknown MA.

### Statistical analysis

The baseline characteristics of the two groups were summarized. Categorical and binary variables are presented in frequencies (*n*) and percentages (%), and *P* values of *χ*^2^ tests (RRID: SCR_001905) are reported for the group differences. Continuous variables are described in means and SD, and *P* values of Wilcoxon rank-sum tests (RRID: SCR_001905) are reported. Pearson correlations (RRID: SCR_001905) among metastasis presence, treatments, and care-setting encounters were estimated.

Total costs under $1 were rounded to $1 before applying a natural logarithm transformation, a common approach for normalizing skewed data distributions. Multivariate linear mixed-effects models (RRID: SCR_015655; refs. 29, 30) assessed fixed effects of metastasis and other factors on proportional pre–post changes in costs, while accounting for random effects from healthcare providers who contributed the largest share of each patient’s costs. Sensitivity analyses excluded patients without treatment intent. All tests were two-sided with significance set at *P* < 0.05. Analyses were performed in R v4.1.2.

### Human studies

The data utilized in this study are de-identified and compliant with Health Insurance Portability and Accountability Act regulations, sourced from Optum’s CDM and provided by IU. As such, its use is exempt from Institutional Review Board oversight.

## Results

### Baseline characteristics

Table 1 summarizes patient baseline characteristics. Among 25,169 new cases, 67.2% were nonmetastatic and 32.8% had metastasis. Compared with non-mCRC patients, those with mCRC were more often male, under 65 years of age, and had commercial insurance and higher incomes but showed lower rates of severe comorbidities. Notably, 26.8% of patients with mCRC had commercial HI compared with 20.9% in the non-mCRC group. Meanwhile, 23.6% of patients with mCRC had the comorbidity index ECI score of 13 or higher versus 27.4% in the non-mCRC group.

**Table 1.** Baseline characteristics of the non-mCRC and mCRC groups.

Characteristic	Non-mCRC (*n* = 16,922)	mCRC (*n* = 8,247)	*P* ^a^
Gender, *n* (%)	​	​	​
Female	8,652 (51.1%)	4,090 (49.6%)	0.022
Male	8,270 (48.9%)	4,157 (50.4%)	​
Race/ethnicity, *n* (%)	​	​	​
White	12,014 (71%)	5,907 (71.6%)	0.307
African American	1,850 (10.9%)	893 (10.8%)	​
Hispanics	1,696 (10%)	759 (9.2%)	​
Asian	543 (3.2%)	271 (3.3%)	​
Unknown	819 (4.8%)	417 (5.1%)	​
Diagnosis age, years [*n* (%)]	​	​	​
Age 45–49	538 (3.2%)	428 (5.2%)	<0.001
Age 50–64	3,299 (19.5%)	1,851 (22.4%)	​
Age 65–74	6,410 (37.9%)	3,057 (37.1%)	​
Age 75+	6,675 (39.5%)	2,911 (35.3%)	​
ECI, *n* (%)	​	​	​
(0) ECI less than 1	3,850 (22.8%)	2,079 (25.2%)	<0.001
(1) ECI 1–5	4,025 (23.8%)	1,997 (24.2%)	​
(2) ECI 6–12	4,405 (26%)	2,223 (27%)	​
(3) ECI 13 or higher	4,642 (27.4%)	1,948 (23.6%)	​
Health insurance type	​	​	​
(0) Commercial insurance	3,535 (20.9%)	2,208 (26.8%)	<0.001
(1) MA only	9,600 (56.7%)	4,305 (52.2%)	​
(2) MA and Medicaid or LIS	2,014 (11.9%)	904 (11%)	​
(3) MA unknown	1,773 (10.5%)	830 (10.1%)	​
Household income group	​	​	​
$100,000 plus	3,431 (20.3%)	1,847 (22.4%)	<0.001
$60,000–<100,000	4,289 (25.4%)	2,118 (25.7%)	​
$40,000–<60,000	3,129 (18.5%)	1,553 (18.8%)	​
Less than $40,000	5,197 (30.7%)	2,273 (27.6%)	​
Unknown	876 (5.2%)	456 (5.5%)	​
Diagnosis year	​	​	​
2017	2,759 (16.3%)	1,237 (15.0%)	<0.001
2018	2,873 (17.0%)	1,472 (17.8%)	​
2019	2,950 (17.4%)	1,425 (17.3%)	​
2020	2,425 (14.3%)	1,329 (16.1%)	​
2021	3,026 (17.9%)	1,458 (17.7%)	​
2022	2,889 (17.1%)	1,326 (16.1%)	​
Pre-CRC costs: mean (SD) in $2023	​	​	​
Pre-CRC medical charges	49,262 ($133,920)	36,734 ($95,410)	<0.001
Pre-CRC medical OOP	953 ($2,611)	872 ($1,396)	0.100
Pre-CRC prescription charges	3,186 ($9,514)	2,813 ($9,605)	<0.001
Pre-CRC prescription OOP	468 ($714)	406 ($664)	<0.001

Abbreviation: CRC, colorectal cancer.

a *P* values of *χ*^2^ tests for categorical variables and of Wilcoxon rank-sum tests for the pre–colorectal cancer costs.

With regard to care costs in the year before colorectal cancer diagnosis, approximately 98% of patients incurred medical service charges and 83% paid OOP. On average, the mCRC group had significantly lower pre–colorectal cancer charges ($36,734, SD = 95,410) compared with the non-mCRC group ($49,262, SD = 133,920), although medical OOP expenses were similar ($872, SD = 1,396 vs. $953, SD = 2,611). The mCRC group also had lower prescription costs than the non-mCRC.

### mCRC, cancer treatment components, and care settings

#### Distribution and correlations among mCRC with treatment components and care settings

**Figure 2.**
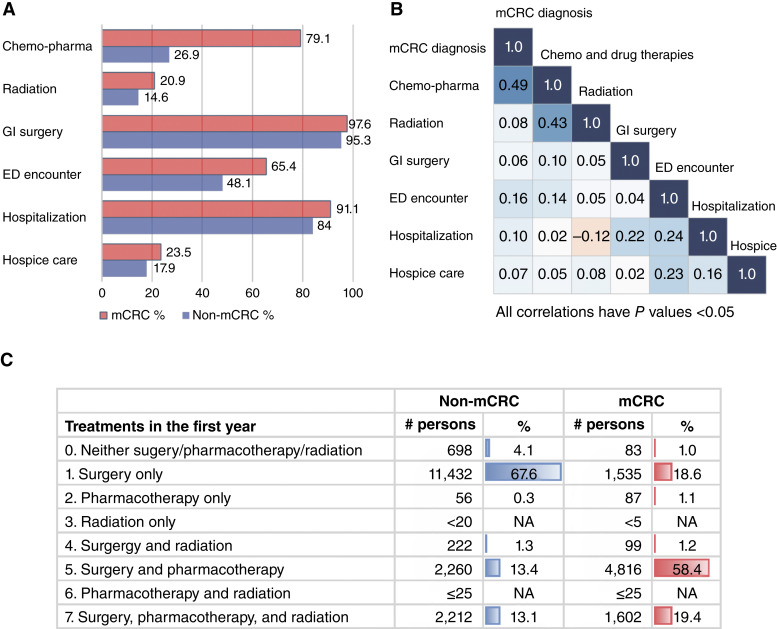
Treatment component, care setting, treatment modality, and metastasis status. **A,** Percent of patients receiving a treatment component and encountering a care setting. **B,** Correlations among metastasis diagnosis, treatment, and care-setting encounters. **C,** First-year treatment modalities for non-mCRC and mCRC patients. Note: small numbers of patients were suppressed in compliance with Optum CDM’s DUA. Chemo-pharma, pharmacotherapy; DUA, data use agreement; NA, not available.

Figure 2 presents the distribution of core treatment components and care-setting encounters in the mCRC and non-mCRC groups, along with estimated correlations, $\hat{\rho }$, between potential cost drivers and mCRC status. Treatment and care utilization were consistently higher in the mCRC group, with the biggest disparity in pharmacotherapy use (79.1% vs. 26.9%), followed by ED visits (65.4% vs. 48.1%), hospitalizations (91.1% vs. 84%), therapeutic radiation (20.9% vs. 14.6%), and hospice care (23.5% vs. 17.9%) in the year following diagnosis (Fig. 2A). Furthermore, metastasis showed the strongest correlation with pharmacotherapy ($\hat{\rho }$ = 0.49), followed by ED visits ($\hat{\rho }=0.16$) and hospitalization ($\hat{\rho }=0.10$), indicating their substantial contribution to cost disparities in metastatic cases (Fig. 2B). With regard to first-year treatment modality, patterns of treatment combination emerged sharpening the picture of intensive pharmacotherapy use for patients with mCRC. Specifically, although most non-mCRC patients underwent surgery alone (67.6%), patients with mCRC predominantly received multimodal therapy, chiefly surgery plus pharmacotherapy (58.4%) or triple-modality treatment with surgery, pharmacotherapy, and radiation (19.4%; Fig. 2C).

#### Post–colorectal cancer medical service charges and OOP expenses

Table 2 summarizes post–colorectal cancer medical charges and OOP costs. On average, patients with mCRC incurred $387,642 in charges (SD = 292,470) and $4,827 in OOP expenses (SD = 4,216) compared with $221,358 (SD = 234,991) and $2,903 (SD = 3,034) for non-mCRC. From before to after colorectal cancer, charges increased by $353,255 for mCRC (SD = 285,923) and $182,000 for non-mCRC (SD = 211,845), with OOP expenses increasing by $4,032 (SD = 4,093) and $2,144 (SD = 2,802), respectively. Half of patients with mCRC saw charge increases of at least $285,826 and OOP increases of $3,054, versus $124,390 and $1,192 for non-mCRC.

**Table 2.** Medical service charges and OOP expenses by core treatment components and care settings (in $2023).

​	Charges			OOP expenses		
	Non-mCRC	mCRC	*P* ^a^	Non-mCRC	mCRC	*P* ^a^
Post-CRC expenses						
Mean (SD)	221,358 (234,991)	387,642 (292,470)	<0.001	2,903 (3,034)	4,827 (4,216)	<0.001
Median (IQR)	157,204 (90,064–276,712)	317,043 (193,561–506,528)	​	2,101 (654–4,143)	4,068 (1,645–7,068)	​
Treatment components^b^						
Nonoperative diagnostic and lab tests						
Mean (SD)	22,526 (23,665)	40,697 (31,976)	<0.001	389 (734)	604 (1,046)	<0.001
Median (IQR)	16,105 (7,646–29,923)	33,455 (18,512–53,536)	​	160 (4–432)	303 (54–674)	​
GI ambulatory surgery						
Mean (SD)	14,438 (14,161)	15,714 (14,939)	<0.001	283 (710)	313 (714)	<0.001
Median (IQR)	11,067 (6,222–18,414)	11,907 (6,672–20,094)	​	0 (0–302)	0 (0–327)	​
Cancer pharmacotherapy						
Mean (SD)	8,179 (33,997)	69,621 (121,256)	<0.001	102 (476)	794 (1,716)	<0.001
Median (IQR)	0 (0–0)	20,852 (0–82,437)	​	0 (0–0)	0 (0–822)	​
Therapeutic radiation						
Mean (SD)	13,687 (42,494)	17,775 (48,438)	<0.001	184 (704)	190 (718)	<0.001
Median (IQR)	0 (0–0)	0 (0–0)	​	0 (0–0)	0 (0–0)	​
Care settings						
Outpatient and not ED						
Mean (SD)	90,298 (114,724)	142,006 (110,166)	<0.001	1,585 (2,124)	2,550 (2,649)	<0.001
Median (IQR)	56,990 (33,375–108,379)	118,927 (70,956–183,258)	​	804 (241–2,038)	1,790 (576–3,747)	​
Inpatient	​	​	​	​	​	​
Mean (SD)	107,585 (148,999)	140,916 (169,989)	<0.001	1,026 (1,452)	1,231 (1,636)	<0.001
Median (IQR)	70,617 (20,537–136,377)	96,541 (45,432–181,786)	​	509 (0–1,606)	700 (0–1,843)	​
ED	​	​	​	​	​	​
Mean (SD)	3,821 (7,518)	6,035 (9,370)	<0.001	37 (155)	46 (171)	<0.001
Median (IQR)	0 (0–4,976)	3,214 (0–7,868)	​	0 (0–0)	0 (0–0)	​
Hospice care						
Mean (SD)	529 (3,540)	836 (5,674)	<0.001	3 (70)	4 (57)	<0.001
Median (IQR)	0 (0–0)	0 (0–0)	​	0 (0–0)	0 (0–0)	​
Pre- and post-CRC difference						
Mean (SD)	182,000 (211,845)	353,255 (285,923)	<0.001	2,144 (2,802)	4,032 (4,093)	<0.001
Median (IQR)	124,390 (55,228–237,673)	285,826 (162,375–471,275)	​	1,192 (0–3,085)	3,054 (688–6,134)	​

For each category that the patient did not encounter, its associated cost was considered as 0.

Abbreviation: CRC, colorectal cancer.

a *P* values of nonparametric Wilcoxon rank-sum tests.

b Diagnostic and lab tests, GI ambulatory surgery, therapeutic radiation, and majority of cancer pharmacotherapy were delivered in the outpatient clinical setting. Cancer pharmaceutical administrations, however, can happen in inpatient setting if a hospital stay was ordered.

By treatment component, patients with mCRC consistently had higher charges. Diagnostic and lab services averaged $40,697 versus $22,526, with median costs of $33,455 versus $16,105; OOP expenses averaged $604 versus $389. Cancer pharmacotherapy showed the largest gap, and average charges were $69,621 versus $8,179, and OOP expenses were $794 versus $102. Although 79.1% of patients with mCRC received pharmacotherapy (50% incurred at least $20,852), only 26.9% of non-mCRC did (Fig. 2). Ambulatory GI surgery and therapeutic radiation also cost more for mCRC than for non-mCRC, averaging $15,714 versus $14,438 and $17,775 versus $13,687, respectively. Additionally, medical service costs by treatment modality, which included combinations of treatment components, are reported in Supplementary Table S6.

Across care settings, outpatient costs for mCRC were 1.6 times higher than for non-mCRC, averaging $142,006 versus $90,298 (medians: $118,927 vs. $56,990), with OOP expenses of $2,550 versus $1,585 (medians: $1,790 vs. $804). Inpatient charges were also higher for mCRC ($140,916 vs. $107,585; medians: $96,541 vs. $70,617), although OOP differences were modest ($1,231 vs. $1,026; medians: $700 vs. $509). ED and hospice charges were greater for mCRC, with minimal OOP variation.

#### Post–colorectal cancer prescription charges and OOP expenses

Table 3 summarizes post–colorectal cancer prescription charges and OOP. Prescription charges increased more sharply for the mCRC group, with a pre–post difference average of $5,745, nearly 2.5 times that of the non-mCRC group ($2,406), and a median of $614, more than seven times that of the non-mCRC ($84). Antineoplastic drugs accounted for more than 60% of this increase in patients with mCRC and more than 40% in non-mCRC. Similarly, average OOP expenses increased by $319 for patients with mCRC, more than 1.5 times the increase seen in non-mCRC ($188), with antineoplastic drugs contributing 45% and 28% of the OOP increases, respectively. Additionally, we also presented these prescription costs for patients undergoing specific treatment modality in the first year in Supplementary Table S7.

**Table 3.** Outpatient pharmacy prescription charges and OOP expenses between patients with mCRC and non-mCRC(in $2023).

​	Charges			OOPS		
	Non-mCRC	mCRC	*P* ^a^	Non-mCRC	mCRC	*P* ^a^
Post-CRC expenses		​	​	​	​	​
Mean (SD)	4,978 (12,139)	7,953 (16,934)	<0.001	542 (789)	624 (949)	<0.001
Median (IQR)	1,050 (190–4,919)	2,120 (351–7,794)	​	290 (87–692)	326 (110–772)	​
Antineoplastic agents						
Mean (SD)	1,112 (5,752)	3,471 (12,199)	<0.001	49 (301)	136 (619)	<0.001
Median (IQR)	0 (0–0)	0 (0–491)	​	0 (0–0)	0 (0–0)	​
Opiate	​	​	​	​	​	​
Mean (SD)	61 (623)	94 (645)	<0.001	17 (83)	24 (97)	<0.001
Median (IQR)	0 (0–10)	3 (0–21)	​	1 (0–6)	2 (0–10)	​
Antibiotics						
Mean (SD)	79 (1,327)	113 (924)	<0.001	22 (130)	25 (102)	<0.001
Median (IQR)	0 (0–11)	1 (0–23)	​	4 (0–14)	5 (0–16)	​
Pre- and Post-CRC difference						
Mean (SD)	2,406 (8,383)	5,745 (15,289)	<0.001	188 (515)	319 (791)	<0.001
Median (IQR)	84 (0–1,321)	614 (0–4,328)	​	5 (0–174)	59 (0–312)	​

For each category that the patient did not encounter, its associated cost was considered as 0.

Abbreviation: CRC, colorectal cancer.

a *P* values of nonparametric Wilcoxon rank-sum tests.

### Adjusted associations of mCRC and other factors with pre–post changes in costs

#### Pre–post changes in medical service charges and OOP expenses

**Figure 3.**
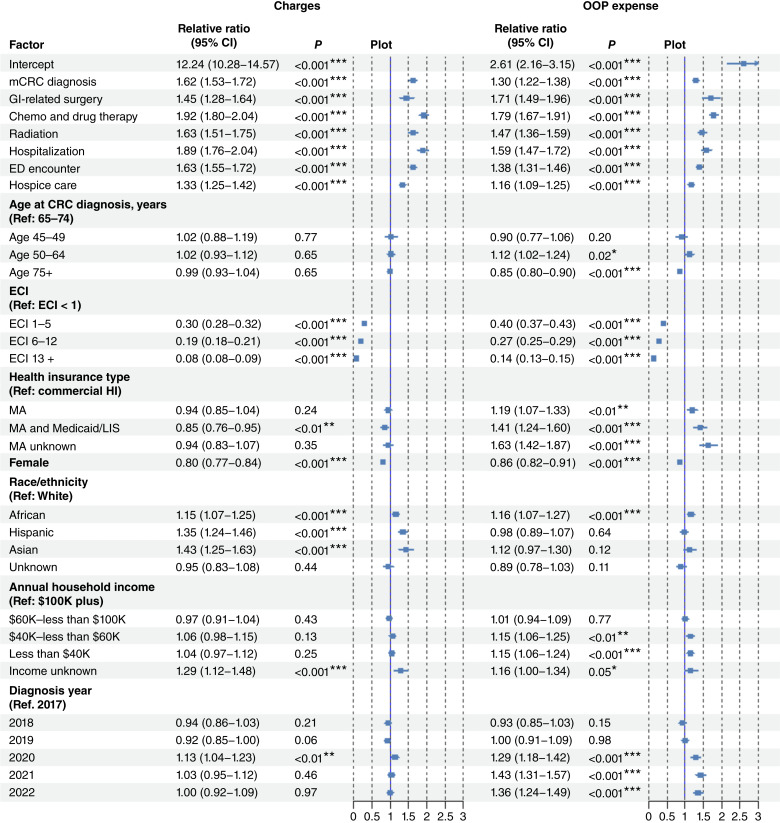
Estimated association of metastasis and other factors with the pre–post proportional increases in medical service charges and OOP expenses. Estimated relative ratio of proportional increase in pre– and post–colorectal cancer (CRC) medical charges and OOP expenses associated with each factor, with 95% CIs, two-sided *P* value, and illustrative plots. Ref, reference. *, *P* < 0.05; **, *P* < 0.01; ***, *P* < 0.001.

Figure 3 presents estimated associations of mCRC status and other factors with proportional changes in medical charges and OOP expenses from before to after diagnosis. The proportional changes (i.e., outcome variable) are defined as the ratio of post–colorectal cancer costs to pre–colorectal cancer costs. On average, patients with mCRC had a 1.6-fold increase in medical charges [95% confidence interval (CI), 1.53–1.72] and a 1.3-fold increase in OOP expenses (95% CI, 1.12–1.28) compared with non-mCRC patients.

Among treatment components, pharmacotherapies were the most impactful, associated with nearly twofold increases in both medical charges and OOP expenses, i.e., 1.9-fold (95% CI, 1.80–2.04) and 1.8-fold (95% CI, 1.64–1.88), respectively. This was followed by radiation with a 1.6-fold increase (95% CI, 1.51–1.75) in medical charges and 1.5-fold (95% CI, 1.38–1.62) in OOP costs and then by GI surgery with 1.4-fold (95% CI, 1.28–1.64) and 1.7-fold (95% CI, 1.08–1.42), respectively.

Across care settings, hospitalizations, ED visits, and hospice care were all significantly associated with greater increases in both medical charges and OOP expenses. Hospitalization emerged as the strongest driver, with 1.9-fold (95% CI, 1.76–2.04) increases in medical charges and 1.6-fold (95% CI, 1.47–1.72) in OOP expenses.

Although diagnosis age was not significantly associated with changes in medical charges, it was associated with OOP expenses. Particularly, patients aged 50 to 64 years paid OOP 1.1 times of those aged 65 to 74 years, whereas those aged 75 years only had 0.9 times. Patients with higher ECI scores experienced smaller increases in both medical charges and OOP expenses compared with those with mild or no comorbidities (see Fig. 3 for category-specific estimates).

Insurance type, gender, race, and income level significantly influence the financial burden of colorectal cancer care. Patients with dual MA and Medicaid/LIS experienced smaller increases in medical charges, whereas those with MA alone paid higher OOP compared with commercially insured individuals. Females generally saw smaller cost increases than males. Racial and ethnic disparities were evident: African American, Hispanic, and Asian patients encountered greater increases in medical charges than White patients, with African Americans also incurring significantly higher OOP expenses. Although household income did not markedly affect changes in medical charges, individuals earning less than $60,000 faced higher OOP increases. Notably, OOPs have consistently increased since 2019.

#### Pre–post changes in prescription charges and OOP expenses

**Figure 4.**
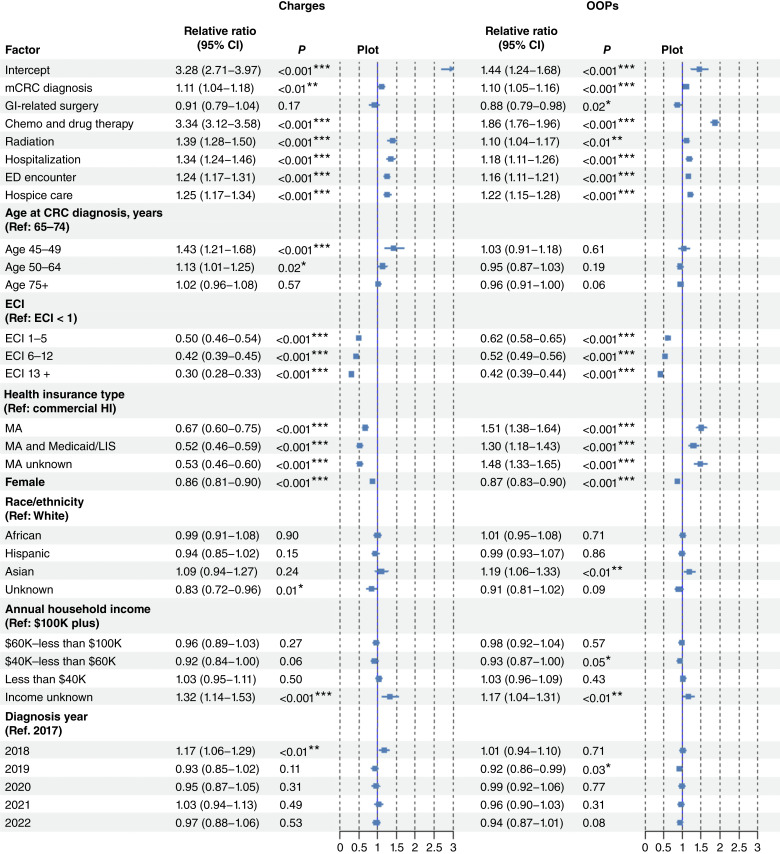
Estimated association of metastasis and other factors with the pre–post proportional increases in outpatient prescription charges and OOP expenses. Estimated relative ratio of proportional increase in pre– and post–colorectal cancer (CRC) prescription charges and OOP expenses associated with each factor, with 95% CIs, two-sided *P* value, and illustrative plots. Ref, reference. *, *P* < 0.05; **, *P* < 0.01; ***, *P* < 0.001.

Figure 4 presents estimated associations of mCRC status and other factors with proportional changes in prescription charges and OOP. mCRC was associated with approximately 10% increase in prescription charges and OOP expenses compared with nonmetastatic cases. Among treatment components, pharmacotherapy had the strongest impact on prescriptions, with total charges and OOP expenses nearly 3.4 and two times those incurred by patients who did not require these therapies. Ambulatory GI surgery was not significantly associated with prescription charges and was modestly linked to lower OOP expenses. Meanwhile, ED, hospitalization, and hospice encounters significantly contributed to increasing these costs although their impact was substantially smaller than that of cancer pharmacotherapy.

Patients under 65 years of age had greater increases in prescription charges but no significant differences in OOP expenses compared with those aged 65 to 74 years. Those with severe ECIs had smaller increases in prescription charges and OOP expenses than those with mild or no comorbidities. Females had lower increases than males, whereas Asians experienced higher OOP increases than Whites. MA enrollees had lower increases in charges but higher in OOP expenses than commercial insurance. Household income and diagnosis years (except years 2018 and 2019) did not play significant roles in prescription costs.

### Sensitivity analysis

We assessed the robustness of the cost changes by excluding individuals who did not have any of the treatment components, resulting in the exclusion of 83 mCRC and 698 non-mCRC patients. The estimated cost increases due to mCRC status and other factors did not change materially (Supplementary Table S8).

## Discussion

This study evaluates the financial burden of mCRC incidents by highlighting the increased healthcare costs associated with mCRC compared with non-mCRC. In our study, 32% of patients had mCRC at diagnosis, exceeding the US average rate of 22.5% estimated by Centers for Disease Control and Prevention for US Cancer Statistics Colorectal Cancer Stat Bite during 2018 to 2022. This difference likely reflects the younger age distribution of our cohort within current epidemiologic trends. Although overall colorectal cancer incidence has declined since the mid-1990s, particularly among seniors (≥65 years; ref. 2), a growing share of new cases over the past two decades has been diagnosed at advanced stages, especially in those <65 years of age (20, 31). Our cohort included patients as young as 45 years, with ages 45 to 49 years comprising 3.8% and ages 50 to 64 years comprising 24.3% of cases (Table 1). Thus, 28% of our study’s patients were aged 45 to 64 years, compared with their 24.6% share of the U.S. population (32). These factors, particularly the younger age distribution, help explain the higher metastatic rate observed relative to earlier reports.

Patients with mCRC had higher rates of hospitalizations and ED visits and faced nearly twice the medical charges and OOP expenses compared with those with non-mCRC, driven by more intensive nonoperative diagnostic and laboratory testing and cancer pharmacotherapy.

The nonoperative diagnostic and lab tests, incurred after the diagnosis and used for staging within the first 6 months and to support initial treatment during the first year, account for substantial costs in both groups, with patients with mCRC facing approximately 80% higher charges than non-mCRC patients. Prior diagnostic estimates for the staging phase were approximately $13,000 per patient per month for stage IV or more than 70% higher than the ∼$7,500 for stages I/II/III during 2000 to 2013 (33). Although the prior estimates were from an earlier period that is not directly comparable with our study, they show a similar trend of steep increase in medical charges of diagnosing and staging for mCRC.

Our study estimated that average medical charges of $353,255 for mCRC exceed those reported in previous studies, which ranged from $12,346 to $300,000 between 2000 and early 2020 (10, 21, 22). Similarly, the average medical OOP expenses across stages (with $4,032 for mCRC and $2,144 for non-mCRC) also surpassed the initial-care average of $2,200 for all cancer types and stages in patients aged ≥65 years with Medicare in 2021 (34).

Several factors likely contribute to these differences. First, our study covers 2017 to 2022, during which advancements in therapeutic interventions, such as robot-assisted surgery, advanced imaging technology, novel targeted agents, and immunotherapies, which are all substantially more costly than traditional procedures, had become widely available. Second, the study includes charges from 2020 to 2022 when inflationary and post–COVID-19 care delivery challenges further increased medical costs (35). Despite these factors, our claims data enabled reliable estimation of patients’ OOP expenses by capturing all components (deductibles, copayments, and coinsurance) within standardized benefit structures, reducing complexity and variability across insurance plans and care providers (36).

The costs distributed across clinical care settings varied between the non-mCRC and patients with mCRC. For non-mCRC patients, inpatient medical charges comprised the largest share of the increases, approximately 59%, whereas for patients with mCRC, inpatient and outpatient charges were nearly equal, each around 40%. Outpatient management, including diagnostic tests, ambulatory GI surgery, cancer pharmacotherapy, and radiation, contributed significantly to both medical charges and OOP expenses.

Given that 77.2% of the cohort was eligible for Medicare, MA plans, which often bundle prescription drug coverage, this enabled more complete tracking of prescription charges and OOP expenses while encouraging the use of lower-cost alternatives when available than the Original Medicare (37). The increase in prescription charges from before to after diagnosis was nearly 2.5 times higher for patients with mCRC than non-mCRC patients. In the mCRC group, antineoplastic drugs accounted for more than 60% of this increase and more than 40% in the non-mCRC group. Prescription OOP expenses followed a similar pattern, with patients with mCRC on average facing more than 1.5 times the OOP increase of non-mCRC patients.

With regard to the proportional cost increases from before to after diagnosis, mCRC was strongly associated with significantly sharper increases in charges and OOP expenses, both in medical service utilizations and prescriptions. However, the most influential cost driver was the pharmacotherapy, which was highly associated with mCRC and nearly doubled medical service charges and OOP expenses, more than tripled prescription charges, and nearly doubled prescription-related OOP expenses. Additionally, care-setting encounters, particularly hospitalization, followed by ED visits and hospice care, were major contributors to cost increases. Our findings also showed an upward trend of medical service OOP expenses since 2019.

Our findings furthermore noted racial and ethnic disparities in financial burden after colorectal cancer diagnosis, with African American patients experiencing sharper proportional increases in OOP expenses for medical services, implying a greater financial hardship following treatment, consistent with prior evidence (38, 39). Meanwhile, Asian American patients showed disproportionately higher increases in OOP prescription costs, which align with prior research documenting barriers to medication access (40) and baseline drug utilization and spending before colorectal cancer diagnosis (41). These results underscore the need for policies that reduce OOP costs and promote equitable access to cancer care for racial minority groups.

Compared with treatment costs for breast cancer, the most prevalent primary invasive cancer in the United States, our findings in colorectal cancer show a similar pattern of increasing expenses with disease progression. In a recent systematic review (42), breast cancer treatment costs for stages I/II/III in the first year after diagnosis were less than half of those for metastatic stage IV during the period 2012 to 2020. In our colorectal cancer analysis, treatment costs similarly escalated with progression, increasing from a median of $124,390 for non-mCRC to $285,826 for mCRC from 2017 to 2023, or approximately a 2.3-fold increase. Thus, although absolute values differ across cancer types and diagnostic or treatment years, the relative cost escalation when moving from nonmetastatic to metastatic disease seems consistent.

By highlighting variations and substantial cost increases linked to metastasized stage and cancer treatments, this study is the first to quantify the financial impact of mCRC from the healthcare system and patient perspectives during the initial-care phase. The findings underscore urgent needs for more efficient care strategies in this early treatment period. Higher costs for patients with mCRC support policies prioritizing early detection, enhancing colorectal cancer care coordination, and improving patient outcomes. Expanding access to preventive services and promoting adherence to screening guidelines may enable earlier diagnoses and help reduce the incidence and, consequently, the economic burden of mCRC.

### Limitations

This study has several limitations. First, the reliance on administrative claims data may introduce biases and limitations inherent to such data sources. Specifically, Optum CDM does not cover uninsured individuals or with only Medicaid or other state-subsidized policy. Second, the absence of detailed clinical information on tumor stage at initial colorectal cancer diagnosis restricted our ability to further disentangle cost differences across specific stages of disease presentation and anticipate its cost progression. Third, the use of billed charges rather than reimbursements may overestimate system costs because of provider-level variation as these charges often reflect confidential, provider-specific discounts that are privately negotiated. To address this, we emphasized proportional differences and adjusted for provider variability using random effects to improve reliability. Future studies using actual reimbursement data are needed for more accurate estimates, particularly for mCRC. Our primary study aim was to estimate and report treatment component, care setting, and other cost driver differences between mCRC and non-mCRC costs. We provided a comparison of our colorectal cancer cost findings with breast cancer costs specifically as it is the most prevalent primary cancer. Future studies should extend comparisons to other high-cost cancers such as lung, prostate, or pancreatic cancer to further contextualize colorectal cancer costs in the broader context of oncology spending trends.

### Conclusion

This study highlights the substantial financial burden faced by newly diagnosed colorectal cancer patients during the initial phase of care, particularly among those with mCRC. The significantly higher costs observed in mCRC cases underscore the importance of early detection and timely intervention. By examining care-cost components and identifying major cost drivers, such as pharmacotherapy, hospitalizations, and emergency care, the findings reveal how late-stage diagnosis amplifies both provider and patient financial strain. These results reinforce the need for policies and programs that promote routine colorectal cancer screening and early diagnosis, especially among high-risk and underserved populations. Enhancing early detection can help reduce the incidence of mCRC, lower treatment intensity, and ultimately improve both clinical outcomes and cost efficiency across the healthcare system.

## Supplementary Material

Table S1 A list of ICD-10-CM diagnosis codes for primary invasive colorectal cancer following case identification and Incident rules of colorectal cancer by AFHSD, and ICD-10-CM diagnosis codes for metastasis cancers by HCUP

Table S2 Types of medical services including facility, auxiliary, and professional services in 3 clinical care settings, whose charges and out-of-pocket payments are elements of the medical service costs

Table S3 Types of medical services for cancer treatment, including ambulatory gastrointestinal surgeries, cancer pharmacotherapy administration, cancer radiation therapy, and non-operative diagnostic testing and laboratory

Table S4 Types of medical services for identifying clinical care settings, including outpatient but not emergency, emergency and inpatient settings

Table S5 Prescription cancer drugs, opiate and antibiotics identified by American Hospital Formular Service Pharmacologic-Therapeutic Classification

Table S6 Estimated differences in medical service costs before and after CRC diagnosis for mCRC vs. non-mCRC patients, stratified by treatment modality during the first year

Table S7 Estimated differences in pharmacy prescription costs before and after CRC diagnosis for non-mCRC versus mCRC patients, stratified by treatment modality in the first year

Table S8 Sensitivity analysis excluding patients who received none of the following: GI surgery, chemotherapy or pharmacotherapy, and radiation therapy

## Data Availability

The data analyzed in this study are available from Optum, Inc. Restrictions apply to the availability of these data, which were used under IU license with Optum for this study. Data are not sharable unless the individual or entity interested in accessing them obtains their Data Use Agreement with Optum.
