# Supplementary material for: Initial-Care Medical and Prescription Costs for Incident Metastatic versus Nonmetastatic Colorectal Cancer
Source: Cancer Res Commun. 2025 Oct 20;5(10):1852–64. doi: 10.1158/2767-9764.CRC-25-0367 (PMC12536409; doi:10.1158/2767-9764.CRC-25-0367)
Supplement: Table S1 — A list of ICD-10-CM diagnosis codes for primary invasive colorectal cancer following case identification and Incident rules of colorectal cancer by AFHSD, and ICD-10-CM diagnosis codes for metastasis cancers by HCUP [file crc-25-0367_table_s1_suppst1.docx]

**Supplement Materials**

**Table S1**: Diagnosis codes for primary invasive colorectal cancer and metastasis cancers

| **ICD-10** | **Description** | **Source** |
| --- | --- | --- |
| Primary invasive colorectal cancer | | |
| C18 | Malignant neoplasm of colon | Case identification and Incident rules of colorectal cancer by [AFHSD](https://health.mil/Reference-Center/Publications/2025/03/01/Colorectal-Cancer) |
| C19 | Malignant neoplasm of rectosigmoid junction |  |
| C20 | Malignant neoplasm of rectum |  |
| C26.0 | Malignant neoplasm of intestinal tract, part unspecified |  |
| Metastasis cancer | | |
| C77.x | Secondary malignant neoplasm of lymph nodes | ICD-10-CM and [HCUP](https://hcup-us.ahrq.gov/toolssoftware/ccsr/dxccsr.jsp) |
| C78.0 | Secondary malignant neoplasm o lung |  |
| C78.1 | Secondary malignant neoplasm of mediastinum |  |
| C78.2 | Secondary malignant neoplasm of pleura |  |
| C78.3 | Secondary malignant neoplasm of respiratory organ |  |
| C78.4 | Secondary malignant neoplasm of small intestine |  |
| C78.5 | Secondary malignant neoplasm of large intestine and rectum |  |
| C78.6 | Secondary malignant neoplasm of retroperitoneum and peritoneum |  |
| C78.7 | Secondary malignant neoplasm of liver and intrahepatic bile duct |  |
| C78.8 | Secondary malignant neoplasm of other or unspecified digestive organ |  |
| C79.0 | Secondary malignant neoplasm of kidney and renal pelvis |  |
| C79.1 | Secondary malignant neoplasm of urinary organs |  |
| C79.2 | Secondary malignant neoplasm of skin |  |
| C79.3 | Secondary malignant neoplasm of brain, cerebral meninges |  |
| C79.4 | Secondary malignant neoplasm of unspecified or other part of nervous system |  |
| C79.5 | Secondary malignant neoplasm of bone |  |
| C79.6 | Secondary malignant neoplasm of ovary |  |
| C79.7 | Secondary malignant neoplasm of adrenal gland |  |
| C79.81 | Secondary malignant neoplasm of breast |  |
| C79.82 | Secondary malignant neoplasm of genital organs |  |
| C79.89 | Secondary malignant neoplasm of other specified sites |  |
| C79.9 | Secondary malignant neoplasm of unspecified site |  |
| C7B.00 | Secondary carcinoid tumors, unspecified site |  |
| C7B.01 | Secondary carcinoid tumors of distant lymph nodes |  |
| C7B.02 | Secondary carcinoid tumors of liver |  |
| C7B.03 | Secondary carcinoid tumors of bone |  |
| C7B.04 | Secondary carcinoid tumors of peritoneum |  |
| C7B.09 | Secondary carcinoid tumors of other sites |  |
| C7B.1 | Secondary Merkel cell carcinoma |  |
| C7B.8 | Other secondary neuroendocrine tumors |  |
| C80.0 | Disseminated malignant neoplasm, unspecified |  |

Notes:

ICD-10-CM the International Classification of Diseases, 10th Revision, Clinical Modification

[AFHSD](https://health.mil/Reference-Center/Publications/2025/03/01/Colorectal-Cancer) the Armed Forces Health Surveillance Division

[HCUP](https://hcup-us.ahrq.gov/toolssoftware/ccsr/dxccsr.jsp) Healthcare Cost and Utilization Project
