# Supplementary material for: Initial-Care Medical and Prescription Costs for Incident Metastatic versus Nonmetastatic Colorectal Cancer
Source: Cancer Res Commun. 2025 Oct 20;5(10):1852–64. doi: 10.1158/2767-9764.CRC-25-0367 (PMC12536409; doi:10.1158/2767-9764.CRC-25-0367)
Supplement: Table S2 — Types of medical services including facility, auxiliary, and professional services in 3 clinical care settings, whose charges and out-of-pocket payments are elements of the medical service costs [file crc-25-0367_table_s2_suppst2.docx]

**Supplement Materials**

**Table S2**: Types of services used to aggregate the medical costs

| Type of medical services | Detailed Description |
| --- | --- |
| *Ancillary medical services and supplies* | |
| Ancillary | Drugs Administered |
| Ancillary | Durable Medical Equipment |
| Ancillary | Home Health/Hospice Visits |
| Ancillary | Services and Supplies |
| Ancillary | Transportation Services |
| *Inpatient (facility and professional) services* | |
| Facility Inpatient | Acute Non-Psych |
| Facility Inpatient | Hospice Facility |
| Facility Inpatient | Rehab/Skilled Nursing Facility |
| Facility Inpatient | Skilled Nursing Facility |
| Facility Inpatient | Long Term Care Non-Acute Care Facility |
| Facility Inpatient | Psych |
| Facility Inpatient | Rehab Facility |
| Professional Services | Inpatient Visits |
| *Emergency department services* | |
| Facility Outpatient | Emergency Department |
| Facility Outpatient | Emergency Room |
| Professional Services | Emergency Department |
| Professional Services | Emergency Room |
| *Outpatient (not including ED) services* | |
| Facility Outpatient | OP Facility Diagnostic |
| Facility Outpatient | OP Facility Laboratory |
| Facility Outpatient | OP Facility Other |
| Facility Outpatient | OP Facility Radiology |
| Facility Outpatient | OP Facility Surgery |
| Professional Services | Allergy Tests and Injections |
| Professional Services | Anesthesia |
| Professional Services | Cellular Therapy |
| Professional Services | Consultations |
| Professional Services | Dental Procedures |
| Professional Services | Diagnostic Testing |
| Professional Services | Immunizations and Injections |
| Professional Services | Laboratory |
| Professional Services | Mental Health |
| Professional Services | Obstetrics |
| Professional Services | Office Visits |
| Professional Services | Pathology |
| Professional Services | Physical Medicine/Rehab |
| Professional Services | Professional Other |
| Professional Services | Preventive Medicine |
| Professional Services | Radiology |
| Professional Services | Surgery |
| Professional Services | Vision, Hearing and Speech |
