# Supplementary material for: Initial-Care Medical and Prescription Costs for Incident Metastatic versus Nonmetastatic Colorectal Cancer
Source: Cancer Res Commun. 2025 Oct 20;5(10):1852–64. doi: 10.1158/2767-9764.CRC-25-0367 (PMC12536409; doi:10.1158/2767-9764.CRC-25-0367)
Supplement: Table S3 — Types of medical services for cancer treatment, including ambulatory gastrointestinal surgeries, cancer pharmacotherapy administration, cancer radiation therapy, and non-operative diagnostic testing and laboratory [file crc-25-0367_table_s3_suppst3.docx]

**Supplement Materials**

**Table S3**: Identification of cancer treatments by types of medical facility, services and procedure codes provided by CDM

| Type of Service – Treatment: Specialty | Description | Type-of-Service in detail |
| --- | --- | --- |
| **Ambulatory GI surgery^(a)^** | | |
| Facility outpatient - Surgery: Digestive | Colonoscopy surgeries, Abdomen or peritoneum or omentum, anus, appendix, biliary tract, esophagus, intestines, liver, meckel and mesentery, pancreas, rectum, stomach | FAC_OP.FO_SUR.DIGEST.CLNSCP, FAC_OP.FO_SUR.AMBSRG.ABDPER, FAC_OP.FO_SUR.DIGEST.ABDPER, FAC_OP.FO_SUR.AMBSRG.ABDOM, FAC_OP.FO_SUR.DIGEST.ABDOM, FAC_OP.FO_SUR.AMBSRG.ANUS, FAC_OP.FO_SUR.DIGEST.ANUS, FAC_OP.FO_SUR.AMBSRG.APPEND, FAC_OP.FO_SUR.DIGEST.APPEND, FAC_OP.FO_SUR.AMBSRG.BILIAR, FAC_OP.FO_SUR.DIGEST.BILIAR, FAC_OP.FO_SUR.AMBSRG.ESOPH, FAC_OP.FO_SUR.DIGEST.ESOPH, FAC_OP.FO_SUR.AMBSRG.INTEST, FAC_OP.FO_SUR.DIGEST.INTEST, FAC_OP.FO_SUR.AMBSRG.LIVER, FAC_OP.FO_SUR.DIGEST.LIVER, FAC_OP.FO_SUR.AMBSRG.MECK, FAC_OP.FO_SUR.DIGEST.MECK, FAC_OP.FO_SUR.AMBSRG.PANCR, FAC_OP.FO_SUR.DIGEST.PANCR, FAC_OP.FO_SUR.AMBSRG.RECT, FAC_OP.FO_SUR.DIGEST.RECT, FAC_OP.FO_SUR.AMBSRG.STOM, FAC_OP.FO_SUR.DIGEST.STOM |
| Professional services - Surgery: Digestive | Abdomen/peritoneum/omentum, anus, appendix, biliary tract, esophagus, intestines, liver, meckel and mesentery, pancreas, rectum, stomach | PROF.SURG.DIGEST.ABDPER, PROF.SURG.DIGEST.ANUS, PROF.SURG.DIGEST.APPEND, PROF.SURG.DIGEST.BILIAR, PROF.SURG.DIGEST.ESOPH, PROF.SURG.DIGEST.INTEST, PROF.SURG.DIGEST.LIVER, PROF.SURG.DIGEST.MECK, PROF.SURG.DIGEST.PANCR, PROF.SURG.DIGEST.RECT, , PROF.SURG.DIGEST.STOM |
| **Cancer Pharmacotherapies^(b)^** | | |
| Ancillary - Drugs administered: Antineoplastic agents | Alkylating agents, nitrogen mustards; anthracycline antibiotics; antibody drug conjugates, chemotherapeut; antiestrogens, chemotherapeutic; antimetabolites; antineoplastic antibiotics, miscellaneou; antineoplastic radiopharmaceuticals; antineoplastics, miscellaneous; biological response modifiers, chemother; chemotherapy adjuncts; cytoprotective agents, chemotherapeutic; gonadotropin releasing hormones, chemoth; mitotic inhibitors, podophyllotoxin deri; mitotic inhibitors, taxanes; mitotic inhibitors, vinca alkaloids; monoclonal antibodies, chemotherapeutic; proteasome inhibitors; topoisomerase inhibitors; vaccines, chemotherapeutic | ANC.DRUGAD.6602 |
| Ancillary - Drugs administered: Cancer therapy | Other cancer therapy | ANC.DRUGAD.CATX |
| Facility outpatient - Other: Cancer therapy | Chemotherapy | FAC_OP.FO_OTH.CHEMO |
| Professional services - Professional other: Cancer therapy | Chemotherapy | PROF.PROOTH.CHEMO |
| **Radiation therapy^(c)^** | | |
| Facility outpatient - Radiology: Therapeutic radiology | Brachytherapy; radionuclide therapy; therapeutic radiology; treatment delivery; treatment management; treatment planning | FAC_OP.FO_RAD.THRRAD.BRACHY, FAC_OP.FO_RAD.THRRAD.NUCLID, FAC_OP.FO_RAD.THRRAD.THRRAD, FAC_OP.FO_RAD.THRRAD.TRTDEL, FAC_OP.FO_RAD.THRRAD.TRTMGT, FAC_OP.FO_RAD.THRRAD.TRTPLN |
| Professional services - Radiology: Therapeutic radiology | Brachytherapy; nuclear medicine; other; radiation oncology; radionuclide therapy; treatment delivery; treatment management; treatment planning | PROF.RAD.THRRAD.BRACHY, PROF.RAD.THRRAD.NUCMED, PROF.RAD.THRRAD.OTHER, PROF.RAD.THRRAD.RADONC, PROF.RAD.THRRAD.NUCLID, PROF.RAD.THRRAD.TRTDEL, PROF.RAD.THRRAD.TRTMGT, PROF.RAD.THRRAD.TRTPLN |
| **Diagnostic testing and laboratory^(d)^** | | |
| Facility outpatient - Diagnostic testing: Allergy testing | Allergy testing | FAC_OP.FO_DIA.ALLTST.ALLTST |
| Facility outpatient - Diagnostic testing: Cardiology | Cardiology, EKG | FAC_OP.FO_DIA.CARD.CARD, FAC_OP.FO_DIA.CARD.EKG |
| Facility outpatient - Diagnostic testing: Ear-Nose-Throat (ENT) | Vestibular function studies | FAC_OP.FO_DIA.ENT.VESTST |
| Facility outpatient - Diagnostic testing: Gastrointestinal services | Gastrointestinal services | FAC_OP.FO_DIA.GASINT.GASINT, FAC_OP.FO_DIA.OTHER.GASTRO |
| Facility outpatient - Diagnostic testing: Other | Biofeedback, neurol, other diagnostics | FAC_OP.FO_DIA.OTHER.BIOFEE, FAC_OP.FO_DIA.OTHER.NEUROL, FAC_OP.FO_DIA.OTHER.OTHER |
| Facility outpatient - Diagnostic testing: Pulmonary function | Pulmonary function, other | FAC_OP.FO_DIA.PULFUN |
| Facility outpatient - Laboratory: Chemistry | Drug assays; drug testing; suppression testing; urinalysis | FAC_OP.FO_LAB.CHEM.DRGASY, FAC_OP.FO_LAB.CHEM.DRGTST, FAC_OP.FO_LAB.CHEM.SUPTST, FAC_OP.FO_LAB.CHEM.URINAL |
| Facility outpatient - Laboratory: Hematology | Transfusion medicine | FAC_OP.FO_LAB.HEMAT.TRANSF |
| Facility outpatient - Laboratory: Immunology | Panels; other | FAC_OP.FO_LAB.IMMUN.PANEL, FAC_OP.FO_LAB.IMMUN.OTHER |
| Facility outpatient - Laboratory: Laboratory | Laboratory | FAC_OP.FO_LAB.LAB.LAB |
| Facility outpatient - Laboratory: Pathology | Pathology | FAC_OP.FO_LAB.PATH.PATH |
| Facility outpatient - Laboratory: Surgical pathology | Surgical pathology | FAC_OP.FO_LAB.SURPTH.SURPTH |
| Facility outpatient - Laboratory: Diagnostic radiology | CT scan; diagnostic/ultrasound; mammography; MRA; MRI; nuclear medicine; PET scan; X-ray; other | FAC_OP.FO_RAD.DIARAD.CTSCN, FAC_OP.FO_RAD.DIARAD.DIAULT, FAC_OP.FO_RAD.DIARAD.MAMMO, FAC_OP.FO_RAD.DIARAD.MRA, FAC_OP.FO_RAD.DIARAD.MRI, FAC_OP.FO_RAD.DIARAD.NUCMED, FAC_OP.FO_RAD.DIARAD.OTHER, FAC_OP.FO_RAD.DIARAD.PETSCN, FAC_OP.FO_RAD.DIARAD.XRAY |
| Professional services - Diagnostic testing: Cardiovascular | Cardiovascular stress test; echocardiography; EKG; electrophysiologic studies; non-invasive vascular; pacemaker; other | PROF.DIAGTS.CARDVS.CARDST, PROF.DIAGTS.CARDVS.ECHOCR, PROF.DIAGTS.CARDVS.EKG, PROF.DIAGTS.CARDVS.ELPHY, PROF.DIAGTS.CARDVS.NINVVS, PROF.DIAGTS.CARDVS.PACEMK. PROF.DIAGTS.CARDVS.OTHER |
| Professional services - Diagnostic testing: Collection of specimen(s) | Collection of specimen(s); handling specimen | PROF.DIAGTS.SPECIM.CLLCT, PROF.DIAGTS.SPECIM.SPEC |
| Professional services - Diagnostic testing: ENT | ENT studies; vestibular function studies | PROF.DIAGTS.ENT.ENTSTD, PROF.DIAGTS.ENT.VESTST |
| Professional services - Diagnostic testing: Gastrointestinal services | Gastrointestinal services | PROF.DIAGTS.OTHER.GASTRO |
| Professional services - Diagnostic testing: Neurological | EEG; electromyography; nerve function testing; neuromuscular testing; other; sleep studies | PROF.DIAGTS.NEUROL.EEG, PROF.DIAGTS.NEUROL.ELMYOG, PROF.DIAGTS.NEUROL.NRVFUN, PROF.DIAGTS.NEUROL.NEURTS, PROF.DIAGTS.NEUROL.OTHER, PROF.DIAGTS.NEUROL.SLPSTD |
| Professional services - Diagnostic testing: Other | Biofeedback, other | PROF.DIAGTS.OTHER.BIOFEE, PROF.DIAGTS.OTHER.OTHER |
| Professional services - Diagnostic testing: Pulmonary | Pulmonary function tests; respiratory therapy | PROF.DIAGTS.PULMO.PULMTS, PROF.DIAGTS.PULMO.RSPTHR, PROF.DIAGTS.PULMO.OTHER |
| Professional services - Laboratory: Chemistry | Drug assays; drug testing; suppression testing; urinalysis; other | PROF.LAB.CHEM.DRGASY, PROF.LAB.CHEM.DRGTST, PROF.LAB.CHEM.PANEL, PROF.LAB.CHEM.SUPTST, PROF.LAB.CHEM.URINAL, PROF.LAB.CHEM.OTHER |
| Professional services - Laboratory: Hematology | Transfusion medicine; other | PROF.LAB.HEMAT.TRANSF, PROF.LAB.HEMAT.OTHER |
| Professional services - Laboratory: Immunology | Panels; other | PROF.LAB.IMMUN.PANEL, PROF.LAB.IMMUN.LAB, PROF.LAB.IMMUN.OTHER |
| Professional services - Laboratory: Microbiology | Microbiology | PROF.LAB.MICROB.MICROB |
| Professional services - Laboratory: Other | Other | PROF.LAB.OTHER.OTHER |
| Professional services - Radiology: Diagnostic radiology | CT scan; diagnostic/ultrasound; mammography; MRA; MRI; nuclear medicine; PET scan; X-ray; other | PROF.RAD.DIARAD.CTSCN, PROF.RAD.DIARAD.MAMMO, PROF.RAD.DIARAD.MRA, PROF.RAD.DIARAD.MRI, PROF.RAD.DIARAD.NUCMED, PROF.RAD.DIARAD.OTHER, PROF.RAD.DIARAD.PETSCN, PROF.RAD.DIARAD.ULTSND, PROF.RAD.DIARAD.XRAY |

*Notes:* These types of services were linked with Current Procedural Terminology (CPT-4) and Healthcare Common Procedure Coding System (HCPCS) as follows.

**^(a)^** *Ambulatory GI surgery* associated with 481 CPT-4/HCPCS procedure codes, including S2083, C9779, 0437T, 0355T, 0184T, 49999, 49906, 49905, 49904, 49900, 49659, 49657, 49656, 49655, 49654, 49653, 49652, 49651, 49650, 49622, 49621, 49596, 49595, 49594, 49593, 49592, 49591, 49590, 49587, 49585, 49572, 49570, 49568, 49566, 49565, 49561, 49560, 49553, 49550, 49525, 49521, 49520, 49507, 49505, 49465, 49460, 49452, 49451, 49450, 49446, 49441, 49440, 49436, 49429, 49424, 49423, 49422, 49421, 49419, 49418, 49412, 49407, 49406, 49405, 49402, 49400, 49329, 49327, 49326, 49325, 49324, 49323, 49322, 49321, 49320, 49255, 49250, 49215, 49205, 49204, 49203, 49185, 49180, 49084, 49083, 49082, 49060, 49040, 49020, 49010, 49002, 49000, 48999, 48153, 48150, 48148, 48140, 48120, 48105, 48102, 47999, 47801, 47785, 47780, 47760, 47715, 47711, 47700, 47610, 47605, 47600, 47579, 47564, 47563, 47562, 47554, 47550, 47540, 47538, 47537, 47536, 47535, 47534, 47533, 47532, 47531, 47490, 47480, 47420, 47399, 47383, 47382, 47381, 47380, 47379, 47370, 47362, 47350, 47300, 47143, 47135, 47130, 47125, 47122, 47120, 47100, 47015, 47010, 47001, 47000, 46999, 46947, 46946, 46945, 46940, 46930, 46924, 46922, 46910, 46900, 46750, 46710, 46706, 46615, 46614, 46612, 46611, 46610, 46608, 46607, 46606, 46604, 46601, 46600, 46505, 46500, 46320, 46288, 46280, 46275, 46270, 46260, 46255, 46250, 46230, 46221, 46220, 46200, 46083, 46080, 46060, 46050, 46040, 46030, 46020, 45999, 45990, 45915, 45910, 45905, 45900, 45805, 45800, 45562, 45560, 45550, 45541, 45520, 45505, 45500, 45499, 45400, 45399, 45398, 45397, 45395, 45393, 45392, 45391, 45390, 45389, 45388, 45386, 45385, 45384, 45382, 45381, 45380, 45379, 45378, 45350, 45349, 45347, 45346, 45342, 45341, 45340, 45338, 45337, 45335, 45334, 45333, 45332, 45331, 45330, 45327, 45320, 45317, 45315, 45309, 45308, 45307, 45305, 45303, 45300, 45190, 45172, 45171, 45160, 45150, 45135, 45130, 45126, 45123, 45121, 45120, 45119, 45114, 45113, 45112, 45111, 45110, 45100, 45020, 45005, 45000, 44979, 44970, 44960, 44955, 44950, 44900, 44899, 44850, 44820, 44800, 44799, 44705, 44701, 44700, 44661, 44660, 44650, 44640, 44626, 44625, 44620, 44615, 44605, 44604, 44603, 44602, 44500, 44408, 44405, 44404, 44403, 44401, 44394, 44392, 44391, 44389, 44388, 44386, 44385, 44382, 44381, 44380, 44379, 44378, 44377, 44376, 44373, 44369, 44366, 44364, 44363, 44361, 44360, 44346, 44345, 44340, 44320, 44316, 44314, 44312, 44310, 44300, 44238, 44227, 44213, 44212, 44211, 44210, 44208, 44207, 44206, 44205, 44204, 44203, 44202, 44188, 44187, 44186, 44180, 44160, 44158, 44157, 44156, 44155, 44151, 44150, 44147, 44146, 44145, 44144, 44143, 44141, 44140, 44139, 44130, 44125, 44121, 44120, 44111, 44110, 44100, 44055, 44050, 44025, 44021, 44020, 44015, 44010, 44005, 43999, 43870, 43860, 43848, 43840, 43830, 43820, 43800, 43775, 43774, 43762, 43761, 43760, 43753, 43752, 43659, 43653, 43644, 43640, 43633, 43632, 43631, 43621, 43611, 43610, 43605, 43499, 43453, 43450, 43332, 43327, 43310, 43289, 43287, 43285, 43282, 43281, 43280, 43279, 43278, 43277, 43276, 43275, 43274, 43273, 43270, 43266, 43265, 43264, 43262, 43261, 43260, 43259, 43255, 43254, 43253, 43252, 43251, 43250, 43249, 43248, 43247, 43246, 43245, 43244, 43243, 43242, 43241, 43240, 43239, 43238, 43237, 43236, 43235, 43233, 43232, 43231, 43229, 43227, 43226, 43220, 43213, 43212, 43202, 43200, 43197, 43196, 43191, 43180, 43130, 43117, 22999, 22905, 22903, 22901, 22900.

**^(b)^** *Chemo and pharmacotherapies* associated with 112 procedure codes including S9331, S9330, S9329, Q5126, Q5123, Q5119, Q5118, Q5117, Q5116, Q5115, Q5112, Q5107, Q2050, Q0084, J9999, J9400, J9395, J9370, J9360, J9358, J9356, J9355, J9351, J9312, J9311, J9310, J9309, J9308, J9306, J9305, J9303, J9299, J9298, J9293, J9280, J9271, J9267, J9264, J9263, J9260, J9250, J9245, J9229, J9228, J9223, J9217, J9216, J9209, J9208, J9206, J9205, J9201, J9200, J9198, J9190, J9185, J9181, J9173, J9171, J9144, J9130, J9100, J9070, J9060, J9055, J9045, J9044, J9041, J9040, J9039, J9035, J9034, J9031, J9030, J9025, J9023, J9022, J9000, J8610, J8521, J8520, J3315, J2783, J1950, J1190, J0894, J0594, G0498, G0070, C9257, A9543, A9529, A9517, 96549, 96542, 96450, 96446, 96425, 96422, 96420, 96417, 96416, 96415, 96413, 96411, 96409, 96406, 96405, 96402, 96401, 96367, 81002.

**^(c)^** *Radiation* associated with 76 procedure codes including Q3001, G6017, G6016, G6015, G6014, G6013, G6012, G6011, G6009, G6005, G6002, G6001, G0340, G0339, C9728, 77799, 77790, 77778, 77772, 77771, 77770, 77620, 77615, 77610, 77605, 77600, 77525, 77523, 77522, 77470, 77469, 77435, 77432, 77431, 77427, 77424, 77423, 77417, 77412, 77407, 77402, 77401, 77399, 77387, 77386, 77385, 77373, 77372, 77371, 77370, 77338, 77336, 77334, 77333, 77332, 77331, 77321, 77318, 77317, 77316, 77307, 77306, 77301, 77300, 77299, 77295, 77290, 77285, 77280, 77263, 77262, 77261, 77014, 74283, 49411, 32701.

**^(d)^** *Diagnostic testing and Laboratory* associated with 2057 CPT-4/HCPCS procedure codes and will be provided upon request.
