# Supplementary material for: Initial-Care Medical and Prescription Costs for Incident Metastatic versus Nonmetastatic Colorectal Cancer
Source: Cancer Res Commun. 2025 Oct 20;5(10):1852–64. doi: 10.1158/2767-9764.CRC-25-0367 (PMC12536409; doi:10.1158/2767-9764.CRC-25-0367)
Supplement: Table S4 — Types of medical services for identifying clinical care settings, including outpatient but not emergency, emergency and inpatient settings [file crc-25-0367_table_s4_suppst4.docx]

**Supplement Materials**

**Table S4**: Identification of care settings by the claimed types of services

| Type of service (TOS) | TOS code | Care setting |
| --- | --- | --- |
| Facility inpatient | FAC_IP | Inpatient |
| Professional services for inpatient visits | PROF.INPVIS | Inpatient |
| Facility outpatient ED/ER | FAC_OP.ED, FAC_OP.ER | ED/ER |
| Professional services in ED/ER | PROF.ED, PROF.ER | ED/ER |
| Facility outpatient, excluding emergency department (ED/ER) | FAC_OP excluding facility types of service used in ED/ER | Outpatient |
| Professional services in outpatient facilities, excluding ED services | PROF excluding professional types of service used in ED/ER | Outpatient |
