# Supplementary material for: Initial-Care Medical and Prescription Costs for Incident Metastatic versus Nonmetastatic Colorectal Cancer
Source: Cancer Res Commun. 2025 Oct 20;5(10):1852–64. doi: 10.1158/2767-9764.CRC-25-0367 (PMC12536409; doi:10.1158/2767-9764.CRC-25-0367)
Supplement: Table S5 — Prescription cancer drugs, opiate and antibiotics identified by American Hospital Formular Service Pharmacologic-Therapeutic Classification [file crc-25-0367_table_s5_suppst5.docx]

**Supplement Materials**

**Table S5**: Identification of prescription drugs related to cancer treatment by types of substance based on American Hospital Formular Service Pharmacologic-Therapeutic Classification ([AHFSCLSS](https://ahfsdruginformation.com/))

| AHFSCLSS | AHFSCLSS Description |
| --- | --- |
| **Cancer drug** |  |
| 10:00.00.00 | Antineoplastic agents |
| **Opiate** |  |
| 28:08.08.00 | Opiate agonists |
| 28:08.12.00 | Opiate partial agonists |
| **Antibiotics** |  |
| 8:12.02.00 | Aminoglycoside antibiotics |
| 8:12.16.08 | Aminopenicillin antibiotics |
| 8:12.18.00 | Quinolone antibiotics |
| 8:12.12.92 | Other macrolide antibiotics |
| 8:12.06.04 | 1st generation cephalosporin antibiotics |
| 8:12.24.00 | Tetracycline antibiotics |
| 8:12.20.00 | Sulfonamide antibiotics (systemic) |
| 8:12.12.04 | Erythromycin antibiotics |
| 8:12.06.12 | 3rd generation cephalosporin antibiotics |
| 8:12.28.20 | Lincomycin antibiotics |
| 8:12.06.08 | 2nd generation cephalosporin antibiotics |
| 8:12.28.16 | Glycopeptide antibiotics |
| 8:12.16.04 | Natural penicillin antibiotics |
| 8:12.12.00 | Erythromycin antibiotics |
| 8:12.07.08 | Carbapenem antibiotics |
| 8:12.28.24 | Oxazolidinone antibiotics |
| 8:12.28.30 | Rifamycin antibiotics |
| 8:12.06.16 | 4th generation cephalosporin antibiotics |
| 8:12.28.12 | Cyclic lipopeptide antibiotics |
| 8:12.06.20 | 5th generation cephalosporin antibiotics |
| 8:12.07.16 | Monobactam antibiotics |
| 8:12.24.12 | Glycylcycline antibiotics |
