# Supplementary material for: Initial-Care Medical and Prescription Costs for Incident Metastatic versus Nonmetastatic Colorectal Cancer
Source: Cancer Res Commun. 2025 Oct 20;5(10):1852–64. doi: 10.1158/2767-9764.CRC-25-0367 (PMC12536409; doi:10.1158/2767-9764.CRC-25-0367)
Supplement: Table S6 — Estimated differences in medical service costs before and after CRC diagnosis for mCRC vs. non-mCRC patients, stratified by treatment modality during the first year [file crc-25-0367_table_s6_suppst6.docx]

**Supplement Materials**

**Table S6:** Estimated pre and post-CRC difference in medical service costs for the non-mCRC versus the mCRC patients, stratified by treatment modality in the first year

|  | Charges | | | OOPs | | | |
| --- | --- | --- | --- | --- | --- | --- | --- |
| **Treatment** | **non-mCRC** | **mCRC** | **P^a^** | **non-mCRC** | **mCRC** | **P^a^** | |
| **Had Neither surgery nor pharmacotherapy nor radiation** | | | | | | | |
| Mean (SD) | 55,381 (151,112) | 55,020 (80,539) | 0.018 | 600 (1,410) | 589 (1,058) | 0.432 | |
| Median (IQR) | 4,504  (0 – 39,268) | 12,776  (0 – 81,021) |  | 0  (0 – 449) | 0  (0 – 775) |  | |
| **Surgery only** | | | | | | | |
| Mean (SD) | 143,641 (185,949) | 193,860 (219,190) | <0.001 | 1,581 (2,120) | 1,844 (2,405) | 0.002 | |
| Median (IQR) | 101,369  (46,413 – 176,716) | 140,477  (74,315 – 245,964) |  | 912  (0 – 2,282) | 1,180  (0 – 2,677) |  | |
| **Pharmacotherapy only** | | | | | | | |
| Mean (SD) | 102,680 (174,679) | 229,961 (229,121) | <0.001 | 1,229 (2,082) | 2,154 (3,049) | 0.085 | |
| Median (IQR) | 22,162  (3,731 – 99,551) | 180,775  (51,602 – 320,485) |  | 8  (0 – 1,788) | 714  (0 – 3,395) |  | |
| **Radiation only** | | | | | | | |
| Mean (SD) | 65,851 (91,440) | 100,175 (57,143) | 0.241 | 849 (1,562) | 2,015 (2,049) | 0.396 | |
| Median (IQR) | 16,521  (0 – 110,307) | 72,846  (67,338 – 119,348) |  | 0  (0 – 449) | 1,948  (974 – 3,022) |  | |
| **Surgery and radiation** | | | | | | | |
| Mean (SD) | 233,371 (226,578) | 302,840 (246,602) | 0.002 | 2,543 (2,755) | 2,769 (2,561) | 0.308 | |
| Median (IQR) | 177,407  (89,662 – 301,672) | 246,598  (143,929 – 369,201) |  | 1,733  (10 – 3,987) | 2,257  (182 – 4,522) |  | |
| **Surgery and pharmacotherapy** | | | | | | | |
| Mean (SD) | 255,609 (223,146) | 373,663 (278,731) | <0.001 | 3,074 (3,430) | 4,468 (4,202) | | <0.001 |
| Median (IQR) | 204,587  (113,692 – 339,000) | 304,810  (189,194 – 488,257) |  | 2,135  (234 – 4,685) | 3,763  (1,145 – 6,613) | |  |
| **Pharmacotherapy and radiation** | | | | | | | |
| Mean (SD) | 175,154 (145,575) | 515,020 (550,601) | <0.001 | 2,843 (3,636) | 4,341 (3,900) | 0.199 | |
| Median (IQR) | 170,425  (43,929 – 257,196) | 377,014  (232,844 – 507,606) |  | 1,622  (0 – 4,241) | 4,494  (20 – 7,356) |  | |
| **Surgery & pharmacotherapy & radiation** | | | | | | | |
| Mean (SD) | 342,814 (236,281) | 468,148 (292,620) | <0.001 | 4,572 (3,737) | 5,175 (4,371) | <0.001 | |
| Median (IQR) | 294,849  (192,273 – 435,275) | 409,724  (276,131 – 583,290) |  | 4,176  (1,628 – 6,566) | 4,648  (1,709 – 7,651) |  | |

Notes:

**^a^** P values of Wilcoxon rank sum tests for the pre-CRC costs.
SD – Standard Deviation; IQR – Inter-Quartile Range; OOP – Out-Of-Pocket expenses
