# Supplementary material for: Initial-Care Medical and Prescription Costs for Incident Metastatic versus Nonmetastatic Colorectal Cancer
Source: Cancer Res Commun. 2025 Oct 20;5(10):1852–64. doi: 10.1158/2767-9764.CRC-25-0367 (PMC12536409; doi:10.1158/2767-9764.CRC-25-0367)
Supplement: Table S7 — Estimated differences in pharmacy prescription costs before and after CRC diagnosis for non-mCRC versus mCRC patients, stratified by treatment modality in the first year [file crc-25-0367_table_s7_suppst7.docx]

**Supplement Materials**

**Table S7:** Estimated pre- and post-CRC difference in pharmacy prescription costs for the non-mCRC the versus mCRC patients, stratified by treatment modality in the first year

|  | Charges | | | OOPs | | | | |
| --- | --- | --- | --- | --- | --- | --- | --- | --- |
| **Treatment** | **non-mCRC** | **mCRC** | **P^a^** | **non-mCRC** | | **mCRC** | | **P^a^** |
| **Neither surgery nor pharmacotherapy nor radiation** | | | | | | | | |
| Mean (SD) | 1,203 (8,449) | 528 (1,540) | 0.167 | 141 (650) | 93 (279) | | 0.236 | |
| Median (IQR) | 0 (0 – 519) | 0 (0 – 210) |  | 0 (0 – 92) | 0 (0 – 46) | |  | |
| **Surgery only** | | | | | | | | |
| Mean (SD) | 1,015 (5,351) | 1,070 (6,279) | 0.396 | 129 (361) | 154 (352) | | 0.018 | |
| Median (IQR) | 14 (0 – 536) | 8 (0 – 485) |  | 0 (0 – 118) | 0 (0 – 143) | |  | |
| **Pharmacotherapy only** | | | | | | | | |
| Mean (SD) | 2,229 (6,851) | 6,160 (30,938) | 0.152 | 157 (403) | 594 (2,249) | | 0.013 | |
| Median (IQR) | 59 (0 – 2,055) | 406 (0 – 3,340) |  | 0 (0 – 130) | 94 (0 – 328) | |  | |
| **Radiation only** | | | | | | | | |
| Mean (SD) | 483 (938) | 415 (711) | 0.728 | 184 (356) | 14 (24) | | 0.55 | |
| Median (IQR) | 0 (0 – 186) | 10 (5 – 623) |  | 0 (0 – 169) | 0 (0 – 21) | |  | |
| **Surgery and radiation** | | | | | | | | |
| Mean (SD) | 1,217 (3,349) | 765 (1,589) | 0.449 | 120 (274) | 190 (410) | | 0.104 | |
| Median (IQR) | 22 (0 – 943) | 0 (0 – 652) |  | 0 (0 – 99) | 0 (0 – 224) | |  | |
| **Surgery and pharmacotherapy** | | | | | | | | |
| Mean (SD) | 5,668 (12,537) | 6,544 (15,661) | 0.008 | 348 (774) | 356 (834) | | 0.579 | |
| Median (IQR) | 803 (0 – 4,570) | 1,023 (34 – 5,192) | | 76 (0 – 335) | 76 (0 – 347) | |  | |
| **Pharmacotherapy and radiation** | | | | | | | | |
| Mean (SD) | 2,761 (5,773) | 17,641 (49,612) | 0.674 | 422 (803) | 1,334 (3,511) | | 0.457 | |
| Median (IQR) | 267 (0 – 2,030) | 195 (16 – 3,224) | | 121 (0 – 401) | 266 (7 – 625) | | | |
| **Surgery & pharmacotherapy & radiation** | | | | | | | | |
| Mean (SD) | 6,775 (12,782) | 8,223 (17,623) | 0.165 | 352 (718) | 356 (714) | | 0.892 | |
| Median (IQR) | 1,515 (197 – 6,912) | 1,813 (171 – 8,759) | | 102 (0 – 396) | 94 (0 – 395) | |  | |

Notes:

**^a^** P values of Wilcoxon rank sum tests for the pre-CRC costs.
SD – Standard Deviation; IQR – Inter-Quartile Range; OOP – Out-Of-Pocket expenses
