# Supplementary material for: Initial-Care Medical and Prescription Costs for Incident Metastatic versus Nonmetastatic Colorectal Cancer
Source: Cancer Res Commun. 2025 Oct 20;5(10):1852–64. doi: 10.1158/2767-9764.CRC-25-0367 (PMC12536409; doi:10.1158/2767-9764.CRC-25-0367)
Supplement: Table S8 — Sensitivity analysis excluding patients who received none of the following: GI surgery, chemotherapy or pharmacotherapy, and radiation therapy [file crc-25-0367_table_s8_suppst8.docx]

**Supplement Materials**

**Table S8:** Sensitivity analysis that excluded those with neither GI surgery, chemo and pharmacotherapies, nor radiation

|  | Medical-service charges | | | Medical-service OOPs | | | | Prescription charges | | | | Prescription OOPs | | | |
| --- | --- | --- | --- | --- | --- | --- | --- | --- | --- | --- | --- | --- | --- | --- | --- |
|  | **RR (95% CI)** | **P** | | **RR (95% CI)** | | **P** | | **RR (95% CI)** | | **P** | | **RR (95% CI)** | | **P** | |
| Intercept | 32.4 (24.36, 43.09) | <0.001 | | 3.71 (2.72, 5.07) | | <0.001 | | 4 (2.9, 5.52) | | <0.001 | | 1.93 (1.51, 2.48) | | <0.001 | |
| **mCRC diagnosis** | 1.6 (1.51, 1.7) | <0.001 | | 1.3 (1.22, 1.38) | | <0.001 | | 1.12 (1.05, 1.19) | | 0.001 | | 1.11 (1.05, 1.16) | | <0.001 | |
| **GI-related Surgery** | 0.54 (0.42, 0.69) | <0.001 | | 1.18 (0.9, 1.56) | | 0.238 | | 0.76 (0.57, 1.01) | | 0.061 | | 0.66 (0.53, 0.82) | | <0.001 | |
| **Chemo & drug therapy** | 1.88 (1.77, 2) | <0.001 | | 1.77 (1.65, 1.89) | | <0.001 | | 3.3 (3.08, 3.54) | | <0.001 | | 1.84 (1.74, 1.94) | | <0.001 | |
| **Therapeutic radiation** | 1.63 (1.51, 1.74) | <0.001 | | 1.47 (1.36, 1.59) | | <0.001 | | 1.39 (1.28, 1.51) | | <0.001 | | 1.11 (1.04, 1.18) | | 0.001 | |
| **Hospital admission** | 1.87 (1.73, 2.02) | <0.001 | | 1.56 (1.44, 1.69) | | <0.001 | | 1.37 (1.26, 1.49) | | <0.001 | | 1.19 (1.11, 1.27) | | <0.001 | |
| **ED encounter** | 1.61 (1.53, 1.69) | <0.001 | | 1.37 (1.3, 1.45) | | <0.001 | | 1.25 (1.18, 1.32) | | <0.001 | | 1.17 (1.12, 1.22) | | <0.001 | |
| **Hospice care** | 1.33 (1.25, 1.41) | <0.001 | | 1.16 (1.08, 1.24) | | <0.001 | | 1.25 (1.17, 1.34) | | <0.001 | | 1.21 (1.15, 1.28) | | <0.001 | |
| **Age at CRC dx**  (Ref: 65-74) | | |  | |  | |  | |  | |  | |  | |  |
| Age 45-49 | 1.05 (0.91, 1.22) | 0.488 | | 0.93 (0.79, 1.09) | | 0.368 | | 1.42 (1.2, 1.67) | | <0.001 | | 1.03 (0.91, 1.17) | | 0.641 | |
| Age 50-64 | 1.04 (0.95, 1.15) | 0.360 | | 1.15 (1.04, 1.28) | | 0.006 | | 1.13 (1.01, 1.25) | | 0.027 | | 0.95 (0.87, 1.03) | | 0.201 | |
| Age 75+ | 0.97 (0.92, 1.03) | 0.298 | | 0.85 (0.8, 0.9) | | <0.001 | | 1.02 (0.96, 1.09) | | 0.450 | | 0.96 (0.91, 1.01) | | 0.106 | |
| **Elixhauser ECI**  (Ref: ECI < 1) | | |  | |  | |  | |  | |  | |  | |  |
| ECI 1 - 5 | 0.32 (0.3, 0.34) | <0.001 | | 0.4 (0.37, 0.43) | | <0.001 | | 0.49 (0.46, 0.53) | | <0.001 | | 0.61 (0.58, 0.65) | | <0.001 | |
| ECI 6 - 12 | 0.2 (0.19, 0.22) | <0.001 | | 0.27 (0.25, 0.29) | | <0.001 | | 0.41 (0.38, 0.45) | | <0.001 | | 0.53 (0.5, 0.56) | | <0.001 | |
| ECI 13 + | 0.09 (0.08, 0.09) | <0.001 | | 0.14 (0.13, 0.15) | | <0.001 | | 0.29 (0.27, 0.32) | | <0.001 | | 0.42 (0.39, 0.44) | | <0.001 | |
| **Insurance type**  (Ref: Commercial HI) | | |  | |  | |  | |  | |  | |  | |  |
| MA | 0.95 (0.86, 1.05) | 0.292 | | 1.22 (1.1, 1.37) | | <0.001 | | 0.66 (0.59, 0.73) | | <0.001 | | 1.5 (1.38, 1.63) | | <0.001 | |
| MA & Medicaid/LIS | 0.85 (0.76, 0.96) | 0.007 | | 1.45 (1.27, 1.64) | | <0.001 | | 0.51 (0.45, 0.57) | | <0.001 | | 1.28 (1.16, 1.42) | | <0.001 | |
| MA unknown | 0.93 (0.82, 1.05) | 0.246 | | 1.69 (1.47, 1.95) | | <0.001 | | 0.51 (0.45, 0.59) | | <0.001 | | 1.47 (1.32, 1.64) | | <0.001 | |
| **Female** | 0.81 (0.77, 0.85) | <0.001 | | 0.86 (0.82, 0.91) | | <0.001 | | 0.86 (0.81, 0.9) | | 0.000 | | 0.87 (0.84, 0.91) | | <0.001 | |
| **Race/Ethnicity**  (Ref: White) | | |  | |  | |  | |  | |  | |  | |  |
| African | 1.16 (1.08, 1.26) | <0.001 | | 1.18 (1.08, 1.28) | | <0.001 | | 1 (0.91, 1.09) | | 0.934 | | 1.02 (0.95, 1.09) | | 0.613 | |
| Hispanic | 1.31 (1.21, 1.43) | <0.001 | | 0.97 (0.88, 1.06) | | 0.452 | | 0.93 (0.84, 1.01) | | 0.099 | | 0.99 (0.92, 1.06) | | 0.771 | |
| Asian | 1.43 (1.25, 1.63) | <0.001 | | 1.12 (0.97, 1.3) | | 0.118 | | 1.11 (0.95, 1.29) | | 0.195 | | 1.22 (1.08, 1.37) | | 0.001 | |
| Unknown | 0.96 (0.84, 1.09) | 0.495 | | 0.9 (0.78, 1.04) | | 0.142 | | 0.83 (0.71, 0.96) | | 0.011 | | 0.91 (0.81, 1.02) | | 0.105 | |
| **Annual HH income** (Ref: $100K plus) | | |  | |  | |  | |  | |  | |  | |  |
| $60K - less than $100K | 0.96 (0.9, 1.03) | 0.273 | | 1.01 (0.94, 1.09) | | 0.711 | | 0.95 (0.88, 1.03) | | 0.199 | | 0.98 (0.92, 1.04) | | 0.458 | |
| $40K - less than $60K | 1.05 (0.97, 1.13) | 0.226 | | 1.15 (1.06, 1.25) | | 0.001 | | 0.92 (0.84, 1) | | 0.050 | | 0.93 (0.87, 0.99) | | 0.029 | |
| Less than $40K | 1.02 (0.95, 1.1) | 0.589 | | 1.15 (1.06, 1.24) | | 0.001 | | 1.03 (0.95, 1.11) | | 0.550 | | 1.02 (0.96, 1.09) | | 0.485 | |
| Income unknown | 1.24 (1.08, 1.42) | 0.002 | | 1.15 (0.99, 1.33) | | 0.062 | | 1.35 (1.16, 1.57) | | <0.001 | | 1.18 (1.05, 1.32) | | 0.007 | |
| **Diagnosis year**  (Ref. 2017) | | |  | |  | |  | |  | |  | |  | |  |
| 2018 | 0.96 (0.88, 1.05) | 0.381 | | 0.94 (0.85, 1.04) | | 0.227 | | 1.17 (1.06, 1.29) | | 0.003 | | 1.01 (0.94, 1.1) | | 0.711 | |
| 2019 | 0.94 (0.87, 1.02) | 0.137 | | 1.01 (0.92, 1.1) | | 0.834 | | 0.92 (0.84, 1) | | 0.063 | | 0.92 (0.86, 0.99) | | 0.022 | |
| 2020 | 1.13 (1.04, 1.23) | 0.004 | | 1.32 (1.2, 1.44) | | <0.001 | | 0.94 (0.86, 1.04) | | 0.221 | | 0.98 (0.91, 1.06) | | 0.667 | |
| 2021 | 1.04 (0.95, 1.12) | 0.412 | | 1.46 (1.33, 1.59) | | <0.001 | | 1.02 (0.93, 1.12) | | 0.612 | | 0.95 (0.89, 1.03) | | 0.201 | |
| 2022 | 0.99 (0.91, 1.08) | 0.858 | | 1.36 (1.24, 1.49) | | <0.001 | | 0.96 (0.87, 1.05) | | 0.352 | | 0.93 (0.86, 1.00) | | 0.038 | |

Notes: The proportional increase is the ratio of post-CRC costs and pre-CRC costs when excluding all patients with neither surgery nor pharmacotherapy nor radiation in the first year;

***, **, * for P<0.001, 0.01 and 0.05, respectively
